# Clinical Features of Preeclampsia Preceded by Fetal Growth Restriction

**DOI:** 10.7759/cureus.51275

**Published:** 2023-12-29

**Authors:** Takayoshi Iijima, Soichiro Obata, Etsuko Miyagi, Shigeru Aoki

**Affiliations:** 1 Perinatal Center for Maternity and Neonate, Yokohama City University Medical Center, Yokohama, JPN; 2 Department of Obstetrics and Gynecology, Yokohama City University Hospital, Yokohama, JPN

**Keywords:** pregnancy, preeclampsia, hypertensive disorders of pregnancy, gestational hypertension, fetal growth restriction

## Abstract

Aim: This study aimed to clarify the perinatal prognosis of preeclampsia (PE) with fetal growth restriction (FGR) and determine appropriate medical interventions for these conditions.

Methods: Singleton births delivered to mothers diagnosed with PE with FGR and hypertension at a tertiary center between January 2010 and June 2021 were included. Only patients with PE were included in the analysis, and patients with superimposed PE were excluded. The FGR-preceding group (group F) included patients who developed FGR first and had elevated blood pressure. The remaining cases were defined as the hypertension-preceding group (group H). The perinatal outcomes between the two groups were then compared. The primary outcome was pregnancy prolongation defined as the time from PE diagnosis to delivery. Secondary outcomes included mode of delivery, maternal outcomes, and neonatal outcomes.

Results: The mean gestational age at the time of PE diagnosis was 34.7 (26-40.1) weeks for group F and 30.3 (22.6-39.4) weeks for group H (P=0.004). The median pregnancy prolongation from the time of PE diagnosis to delivery was eight (2-30) days in group F and 10.5 (2-43) days in group H, with no significant difference (P=0.52). The incidence of maternal critical complications was 10.4% in group F and 28.1% in group H (P=0.03; odds ratio 3.36; 95% confidence interval 1.13-10).

Conclusions: Among patients with PE, group H was more likely to develop serious maternal complications than group F, suggesting different pathogenesis between these types of PE. Both groups required cautious perinatal management, but more stringent maternal management was required for group H.

## Introduction

Hypertensive disorders of pregnancy (HDP) are serious complications; among them, preeclampsia (PE) is estimated to occur in 4.6% (2.7-8.2%) of all pregnancies [1]. The prognosis of PE is poor, with increased risk of maternal complications such as pulmonary edema, acute renal failure, cerebrovascular disease, and disseminated intravascular coagulation (DIC) [2]. The International Society for the Study of Hypertension in Pregnancy (ISSHP) revised the classification of HDP in 2014 [3] with the addition of uteroplacental dysfunction, which includes fetal growth restriction (FGR), to the diagnostic criteria for PE. Since the revision, patients with gestational hypertension (GH) and accompanying FGR are considered patients with PE. Neonatal mortality in patients with PE with FGR is significantly higher than that in those without FGR, with no difference in maternal outcomes [4]. Moreover, the frequency of adverse maternal outcomes in PE with FGR is higher than that in PE without FGR [5]. In spite of this, the American College of Obstetricians and Gynecologists (ACOG) does not include FGR in the diagnostic criteria of PE as the management of PE with FGR and PE without FGR is similar [6]. In our previous study, we reported that many cases of PE with hypertension and FGR were able to prolong pregnancy without other organ complications [7].

Numerous studies have explored PE accompanied by FGR; however, the timing of FGR onset in PE cases has never been investigated. Therefore, patients who present with FGR before developing hypertension and those who present with FGR after developing hypertension or concurrently with hypertension are all equally diagnosed with PE. Therefore, this study aimed to clarify the perinatal prognosis of PE with FGR, which is a newly added diagnostic parameter, and to determine the appropriate medical interventions.

This article was previously presented as a meeting abstract at the 74th Annual Meeting of the Japan Society of Obstetrics and Gynecology on August 7, 2022.

## Materials and methods

**Figure 1:**
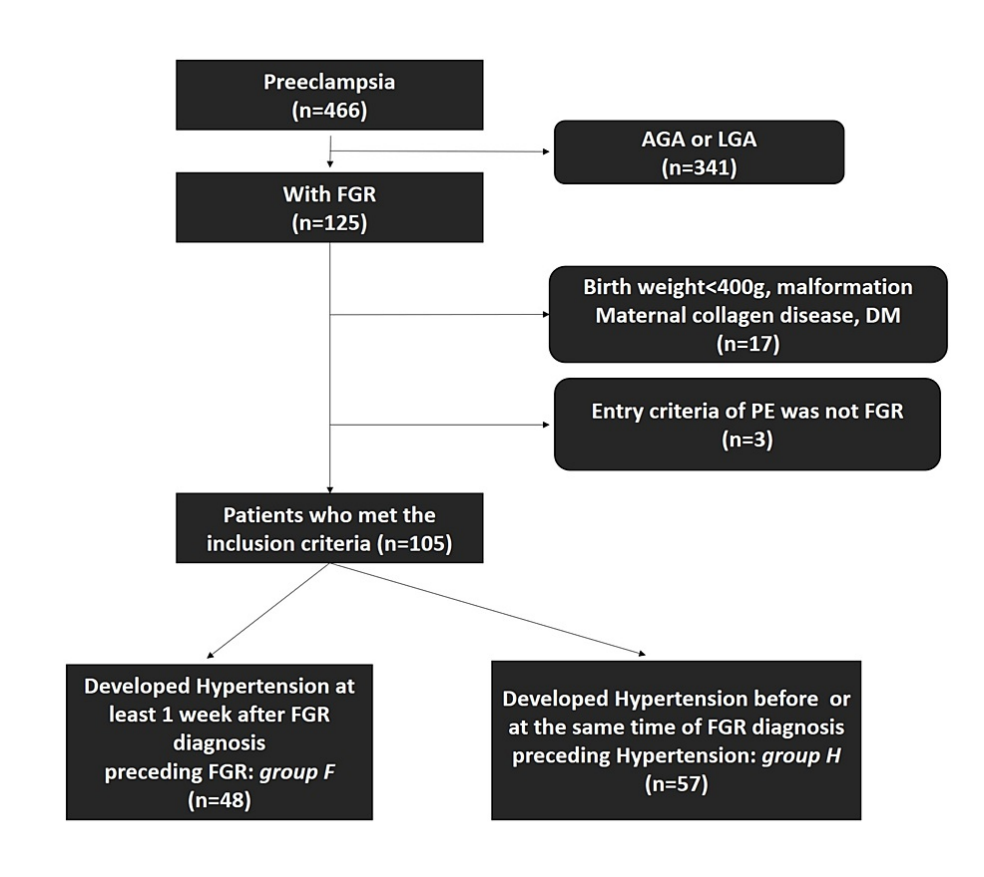
Flowchart of the study protocol. Group F: preceding FGR group; group H: preceding hypertension group; AGA: appropriate for gestational age; LGA: large for gestational age; FGR: fetal growth restriction; DM: diabetes mellitus; PE: preeclampsia; GH: gestational hypertension

Among cases of singleton pregnancies with PE that were seen between January 2010 and June 2021 at the Yokohama City University Medical Center, Yokohama, Japan, those with FGR as an entry criterion for PE diagnosis were included in this study (Figure 1).

For cases prior to 2014, we also used the ISSHP's revised diagnostic criteria for PE by reviewing detailed medical records. We selected all of the hypertension cases and reviewed whether or not the patient in each case had FGR. All cases diagnosed as PE by the current diagnostic criteria were selected, even if they had not been diagnosed as PE by the previous criteria. According to the revised diagnostic criteria of ISSHP, PE was defined as follows: new-onset hypertension (≥140/90 mmHg) after 20 weeks' gestation and symptoms of (1) proteinuria (≥300 mg/day or urine protein/creatinine ≥0.3), (2) renal insufficiency (creatinine >1 mg/dL), (3) liver involvement without underlying disease (aspartate aminotransferase (AST) >40 IU/L or alanine aminotransferase (ALT) >40 IU/L, right upper quadrant or epigastric abdominal pain), (4) neurological complications (eclampsia, cerebral hemorrhage, stroke, severe headache), (5) hematological complications (platelet count <150,000/dL, DIC, hemolysis), and (6) uteroplacental dysfunction (FGR, abnormal umbilical artery Doppler waveform analysis, or intrauterine fetal death (IUFD)) [3]. FGR was defined as an ultrasound-estimated fetal weight ≤-1.5 SD. Fetal weight was based on the "Standard values of ultrasonic measurements in Japanese fetuses" of the Japan Society of Ultrasonics in Medicine [8]. Cases with a diagnosis of FGR on multiple ultrasounds and an estimated fetal weight ≤-1.5 SD on the final ultrasound before delivery were selected. Patients with FGR diagnosed during pregnancy but with birth weights above the 10th percentile were excluded. Other exclusion criteria were fetal malformation/chromosomal abnormalities, maternal collagen disease, and diabetes mellitus (DM). We also excluded the cases in which birth weight was less than 400 g, since we do not decide termination with an estimated fetal weight of less than 400 g by fetal indication at our center. Some cases did not receive neonatal resuscitation because of the extreme prematurity.

All patients with PE were admitted to the hospital for management from the time of diagnosis until delivery. Maternal blood pressure was measured a minimum of three times per day. Blood and urine samples were collected at least once a week on and after admission at the discretion of the attending physician. Daily non-stress tests (NSTs) were performed, and biophysical scores were measured twice per week when needed. Fetal weight and amniotic fluid volume were assessed by abdominal ultrasound at least twice a week. Betamethasone (12 mg intramuscular injection two doses, 24 hours apart) was administered to patients with a gestational age <34 weeks if delivery was expected within one week. If delivery was expected within 24 hours for patients at <32 weeks of gestation, a single intravenous dose of 4 g magnesium sulfate was administered to reduce the risk of cerebral palsy in newborns.

Patients with severe hypertension (≥160/110 mmHg) were administered nifedipine, methyldopa, and labetalol at the discretion of the attending physician. When oral medication was insufficient to reduce blood pressure, or blood pressure was ≥180/120 mmHg, intravenous nicardipine was administered as an emergency antihypertensive therapy. Magnesium sulfate was administered when nicardipine was needed to control blood pressure when it exceeded 160/110 mmHg at delivery or when there were clinical symptoms such as eclampsia or severe headache.

In all patients, we attempted to prolong pregnancy up to 37 weeks, and delivery was only induced after 37 weeks of gestation. In cases of PE diagnosed after the 37th week of gestation, delivery was initiated. Similarly, delivery was induced in patients with PE with severe hypertension after 34 weeks.

Delivery was indicated during pregnancy prolongation when the following symptoms were detected [9]: (1) blood pressure ≥160/110 mmHg despite adequate antihypertensive medication; (2) eclampsia, hypertensive encephalopathy, and severe headache; (3) hemolysis, elevated liver enzymes, and low platelets (HELLP) syndrome, progressive liver dysfunction, and progressive thrombocytopenia; (4) pulmonary edema; (5) acute renal failure (base serum creatinine +1 mg/dL); or (6) placental abruption. HELLP syndrome was diagnosed when all the following symptoms were present: (1) hemolysis (total bilirubin >1.2 mg/dL or lactate dehydrogenase (LDH) >600 IU/L), (2) elevated liver enzymes (AST >70 IU/L), and (3) thrombocytopenia (platelet count <100,000/µL).

Fetal indications included (1) fetal heart rate abnormality (repeated late decelerations or severe variable decelerations in traditional NSTs), (2) biophysical profile score ≤4, and (3) reversal of diastolic umbilical artery blood flow after 32 weeks. IUFD was induced after diagnosis. For the analysis of gestational prolongation, IUFD and deliveries that could not wait for more than 48 hours were excluded.

In this study, patients with PE patients with FGR were divided into two groups: group F wherein FGR diagnosis preceded PE diagnosis by at least one week and group H wherein GH diagnosis preceded FGR. In all cases, FGR was the entry criterion for PE. Other causes of organ damage (including proteinuria) developed at the same time or after diagnosis of FGR. Maternal complications also developed at the same time or after PE diagnosis or during the postpartum period.

This study was approved by the Ethics Committee of Yokohama City University Medical Center (approval number: F211200008). As this was a retrospective study, the Ethics Committee permitted the use of the opt-out method for obtaining informed consent from the patients.

Outcomes

The primary outcome in this study was pregnancy prolongation, which was defined as the time from PE diagnosis to delivery. The secondary outcomes were the mode of delivery, maternal outcomes, and neonatal outcomes. Critical maternal complications were defined as follows: (1) maternal death, (2) eclampsia or cerebral hemorrhage/infarction, (3) pulmonary edema, (4) HELLP syndrome, (5) placental abruption, (6) DIC, and (7) acute renal failure (base serum creatinine +1 mg/ dL). Other maternal complications were defined as proteinuria (≥300 mg/day or urine protein/creatinine ≥0.3), liver involvement (AST >40 IU/L or ALT >40 IU/L), and hematological complications (platelet count <150,000/μL). The timing of onset of maternal complications, as in gestational weeks or postpartum period, was also investigated.

Neonatal outcomes were compared according to birth weight, acidosis (defined as UA pH <7.1), Apgar score at 5 min <7, and neonatal intensive care unit (NICU) admission. Severe adverse neonatal outcomes were defined as follows: (1) perinatal death, (2) necrotizing enterocolitis (NEC), (3) intraventricular hemorrhage 3-4°, (4) periventricular leukomalacia, (5) chronic lung disease, and (6) sepsis. Cases of early-onset PE (diagnosed at <34 weeks) were also analyzed.

Statistics

The data obtained are presented as median (maximum-minimum) and numbers (frequency). We compared continuous variables using the Mann-Whitney U test and categorical variables using Fisher's exact test. In this study, P<0.05 was set as the significance level. Statistical analysis was performed using the JMP Pro 15 (JMP Statistical Discovery LLC, Cary, North Carolina, United States).

## Results

**Figure 2:**
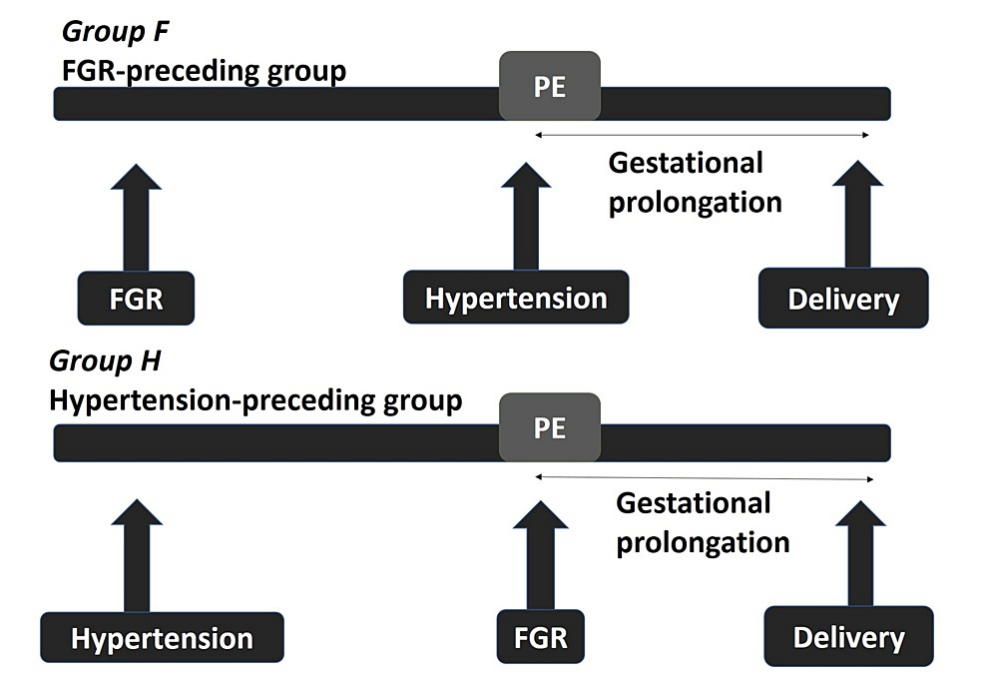
The definition of group F and group H. Group F developed FGR first. Group H developed hypertension first. FGR: fetal growth restriction; PE: preeclampsia

During the study period, a total of 466 singleton births were delivered to mothers diagnosed with PE, of which 125 were accompanied by FGR. We excluded 17 patients with fetal birth weights <400 g, malformations, maternal collagen disease, or DM. Three patients were excluded for having entry criteria for PE other than FGR and developing FGR afterward. We enrolled 105 PE patients with hypertension and FGR in the analysis. Of these, 48 were in the FGR-preceding group (group F), with FGR having been diagnosed at least one week before developing hypertension, and 57 were in the hypertension-preceding group (group H), with hypertension having developed before or simultaneously with FGR diagnosis (Figure 1 and Figure 2).

Maternal backgrounds are shown in Table 1. There were no significant differences in age at delivery, pre-pregnancy body mass index (BMI), BMI at delivery, or primiparity rate. However, the median gestational age upon PE diagnosis was significantly earlier in group H than in group F (30.3 vs 34.7 weeks, P=0.004). The median number of days from the diagnosis of hypertension to FGR onset in group H was 0 days (0-80 days). Similarly, the median number of days from the diagnosis of FGR to the onset of hypertension in group F was 7 days (7-127 days).

**Table 1:** Maternal characteristics. F: fetal growth restriction-preceding group; H: hypertension-preceding group; BMI: body mass index; PE: preeclampsia

Variables	F (n=48)	H (n=57)	Total (n=105)	p-value	Odds ratio	95% CI
Maternal age (years), median (range)	34.5 (18-45)	35 (25-43)	35 (18-45)	0.54		
Pre-pregnancy BMI, median (range)	21.6 (16.5-32.4)	20.8 (16-31.5)	21 (16-32.4)	0.87		
BMI at delivery, median (range)	24.4 (16.8-35.3)	24.5 (18.2-36.4)	24.4 (16.8-36.4)	0.67		
Nulliparous, n (%)	31 (64.6)	34 (59.7)	65 (61.9)	0.69	0.81	0.37-1.79
Gestational age at diagnosis of PE (weeks), median (range)	34.7 (26-40.1)	30.3 (22.6-39.4)	32.7 (22.6-40.1)	<0.01		
Early-onset PE, n (%)	23 (47.9)	40 (70.2)	63 (60)	0.03	2.56	1.15-5.56

Table 2 shows the pregnancy outcomes and maternal complications of the two groups. The proportion of patients who had more than two days of pregnancy prolongation from PE diagnosis to delivery was 72.3% in group F and 84.2% in group H, with no significant difference (P=0.16, odds ratio (OR) 2.04; 95% confidence interval (CI) 0.78-5.31). The median pregnancy prolongation was not significantly different between groups F and H (8 vs 10.5 days, P=0.52). Similarly, the rates of cesarean delivery and pregnancy termination indicated for non-reassuring fetal status (NRFS) were not significantly different. The incidence of maternal critical complications was 10.4% in group F and 28.1% in group H (P=0.03; OR 3.36; 95% CI 1.13-10). Pulmonary edema and HELLP syndrome occurred at a rate of 2.1% in group F and 12.3% in group H (P=0.07, OR 6.58; 95% CI 0.78-55.5). Of the 105 patients, maternal neurological complications occurred in only one case in group H. Proteinuria was observed in 29 patients (60.4%) in group F and 46 patients (80.7%) in group H (P=0.03, OR 2.74; 95% CI 1.14-6.58). There were no differences in the rates of liver involvement or hematological complications. The onset of the organ damage and the PE diagnosis was simultaneous in 15/48 cases (31.3%) in group F and 31/57 cases (54.4%) in group H. Among the cases in the group F, 13 (27.1%) developed organ damage after PE diagnosis. Among the group H, 16 cases (28.1%) developed organ damage after PE diagnosis.

**Table 2:** Maternal outcomes. F: fetal growth restriction-preceding group; H: hypertension-preceding group; BMI: body mass index; NRFS: non-reassuring fetal status; HELLP: hemolysis, elevated liver enzymes, and low platelets; DIC: disseminated intravascular coagulation Note: Critical maternal complications were defined as neurological complications, pulmonary edema, HELLP syndrome, placental abruption, DIC, and renal insufficiency

Variables	F (n=48)	H (n=57)	Total (n=105)	p-value	Odds ratio	95% CI
Gestational age at delivery (weeks), median (range)	35.6 (27.1-40.3)	32 (24.1-39.6)	34 (24.1-40.3)	0.006		
Pregnancy prolongation ≧2 days, n (%)	34 (72.3)	48 (84.2)	82 (78.8)	0.16	2.04	0.78-5.31
Pregnancy prolongation (days), median (range)	8 (2-30)	10.5 (2-43)	9 (2-43)	0.52		
Cesarean delivery, n (%)	32 (66.7)	42 (73.7)	74 (71.2)	0.52	1.40	0.60-3.25
NRFS (indication for delivery), n (%)	16 (34)	24 (42.1)	40 (38.5)	0.43	1.41	0.63-3.14
Induced preterm delivery or scheduled cesarean delivery, n (%)	9 (18.8)	7 (12.3)	16 (15.2)	0.42	0.61	0.21-1.77
Proteinuria, n (%)	29 (60.4)	46 (80.7)	75 (71.4)	0.03	2.74	1.14-6.58
Liver involvement, n (%)	6 (12.5)	10 (17.5)	16 (15.2)	0.59	1.49	0.50-4.45
Hematological complications, n (%)	13 (27.1)	12 (21.1)	25 (23.8)	0.5	0.72	0.29-1.77
Maternal critical complications, n (%)	5 (10.4)	16 (28.1)	21 (20)	0.03	3.36	1.13-10
Neurological complications, n (%)	0 (0)	1 (1.8)	1 (1)	1	-	-
Pulmonary edema, n (%)	1 (2.1)	7 (12.3)	8 (7.6)	0.07	6.58	0.78-55.52
HELLP syndrome, n (%)	1 (2.1)	7 (12.3)	8 (7.6)	0.07	6.58	0.78-55.52
Placental abruption, n (%)	4 (8.3)	4 (7)	8 (7.6)	1	0.83	0.20-3.51
DIC, n (%)	1 (2.1)	1 (1.8)	2 (1.9)	1	0.84	0.05-13.79
Renal insufficiency, n (%)	1 (2.1)	0 (0)	1 (1)	0.46	-	-

Table 3 shows the neonatal outcomes in both groups. Medians of the birth weights were 1,638 g (518-2,492 g) in group F and 1,280 g (444-2,608 g) in group H (P=0.12). The rate of umbilical artery pH <7.1 was 9.5% in group F and 11.3% in group H, with no significant difference between the two groups. Low Apgar score rates (<7 at five minutes) were higher in group H than those in group F (21.3% vs 40.4%), but the difference was not significant (P=0.06, OR 2.50; 95% CI 1.04-6.01). Out of 105 patients, there was only one IUFD case in group F, and no neonatal deaths occurred in either group. NICU admission rates did not differ between the two groups. Severe adverse neonatal outcomes were 17% in group F and 26.3% in group H, which was not significantly different (P=0.34, OR 1.74; 95% CI 0.67-4.56).

**Table 3:** Neonatal outcomes. F: fetal growth restriction-preceding group; H: hypertension-preceding group; NICU: neonatal intensive care unit; NEC: necrotizing enterocolitis; IVH: intraventricular hemorrhage; PVL: periventricular leukomalacia; CLD: chronic lung disease Note: Severe adverse neonatal outcomes were defined as stillbirth, neonatal death, NEC, IVH 3-4°, PVL, CLD, and sepsis

Variables	F (n=48)	H (n=57)	Total (n=105)	p-value	Odds ratio	95% CI
Birth weight (grams), median (range)	1638 (518-2492)	1280 (444-2608)	1426 (444-2608)	0.12		
Umbilical artery pH <7.1, n (%)	4/42 (9.5)	6/53 (11.3)	10 (10.5)	1	1.21	0.32-4.61
Apgar score at 5 min <7, n (%)	10 (21.3)	23 (40.4)	33 (31.7)	0.06	2.50	1.04-6.01
NICU admission, n (%)	36 (76.6)	42 (73.7)	78 (75)	0.82	0.86	0.35-2.10
Severe adverse neonatal outcomes, n (%)	8 (17)	15 (26.3)	23 (22.1)	0.34	1.74	0.67-4.56
Stillbirth, n (%)	1 (2.1)	0 (0)	1 (1)	0.46	-	-
Neonatal death, n (%)	0 (0)	0 (0)	0 (0)	-	-	-
NEC, n (%)	0 (0)	2 (3.5)	2 (1.9)	0.5	-	-
IVH3-4, n (%)	0 (0)	0 (0)	0 (0)	-	-	-
PVL, n (%)	0 (0)	1 (1.8)	1 (1)	1	-	-
CLD, n (%)	6 (12.8)	14 (24.6)	20 (19.2)	0.14	2.22	0.78-6.34
Sepsis, n (%)	1 (2.1)	4 (7)	5 (4.8)	0.37	3.47	0.37-32.18

## Discussion

Among the patients with PE diagnosed with hypertension and FGR, we found two important points. First, in group F, FGR preceded PE, and the patients delivered significantly later compared with those in group H. Second, the incidence of critical maternal complications was significantly higher in group H than in group F. There were no differences in neonatal outcomes or complications between groups.

Takahashi et al. reported that the median gestational week at PE diagnosis was 31.6 (±4.6) weeks for patients diagnosed with PE due to placental dysfunction, including FGR [10]. In this study, the median number of gestational weeks at which PE was diagnosed in all patients was 32.7 (±4.6). In groups F and G, it was 34.7 and 30.2 weeks, respectively (P<0.01). Previous studies have reported an association between disorders of deep placentation and obstetric syndromes, including PE and FGR [11-13]. Uteroplacental dysfunction makes up 25-30% of FGR causes and is the most common cause other than fetal factors [14]. Uteroplacental dysfunction includes umbilical cord abnormalities such as umbilical cord attachment site abnormality, velamentous cord insertion, and a single umbilical cord artery. The causes of FGR are diverse and often overlap with each other. In addition to uteroplacental factors, its causes include fetal chromosomal abnormalities; genetic diseases; congenital infections of toxoplasmosis, others (syphilis, hepatitis B), rubella, cytomegalovirus (CMV), and herpes simplex (TORCH) syndrome; maternal nutritional disorders; smoking; and other drug use. Although fetal malformations and chromosomal abnormalities were excluded from this study, a certain percentage of FGR cases caused by factors that were not placental might have been included. Moreover, healthy but constitutionally small infants with a low risk of perinatal morbidity and mortality may have been included among patients diagnosed with FGR. In such patients, the mechanism by which blood pressure increases after the onset of FGR may be different. Although it is important to manage them with attention to maternal and fetal well-being, these patients should be managed as having different pathophysiological backgrounds.

Second, the incidence of serious maternal complications was significantly higher in group H; however, there was no difference in the incidence of neonatal complications. Mitani et al. reported a 20% incidence of serious maternal complications in patients with PE with FGR [5]. Similar to previous reports, the incidence of serious maternal complications was also 20% in this study. There was a significant difference between groups F and H, indicating that a certain number of patients with PE diagnosed before FGR had different pathologies from other patients with PE. Given the high possibility of serious maternal complications in patients with GH followed by FGR, more rigorous maternal monitoring, including blood tests, and careful post-delivery maternal management are required. However, both PE and FGR have been reported to be independent risk factors for placental abruption [15,16], and in this study, the incidence of placental abruption was 8.3% in group F and 6.7% in group H; this difference was not statistically significant, indicating that careful management is still required in group F.

The rate of severe adverse neonatal outcomes in this study was 17% in group F and 26.3% in group H (P=0.34, OR 1.74; 95% CI 0.67-4.56). Weiler et al. reported eight stillbirths (11.3%), 15 perinatal deaths (22.1%), and three NEC (4%) among 68 patients with severe early-onset PE with FGR [4]. In our analysis, there was only one stillbirth (1.6%) and no neonatal deaths among early-onset PE. These were lower than those in previous reports, and all patients were strictly managed as inpatients after the diagnosis of PE, which facilitated the delivery of the infant at an appropriate time. A patient was admitted to our hospital at 23 weeks' gestation with FGR, and the absence of umbilical artery end-diastolic flow was detected on admission. At 26 weeks of gestation, she was diagnosed with PE with reversed umbilical artery end-diastolic flow. The patient was managed cautiously, but fetal death occurred at 27 weeks and three days.

This study had two limitations. First, it was a retrospective study with a small number of patients. However, this was a single-center study which allowed for a detailed review of medical records. Second, this was an 11-year analysis, with slight differences in delivery criteria due to changes in diagnostic criteria for PE and staff changes during the study period.

## Conclusions

This study showed that patients in whom FGR preceded PE diagnosis had longer gestational ages than patients in whom GH diagnosis preceded FGR. It should be recognized that FGR preceding PE and hypertension preceding PE are both eventually diagnosed as PE, yet they follow different clinical courses and have different underlying pathophysiologies. Perinatal management should be tailored to individual conditions, and, considering that PE with hypertension followed by FGR is more likely to result in serious maternal complications, more stringent prenatal and postnatal maternal management is needed.
